# Developing Brain Vital Signs: Initial Framework for Monitoring Brain Function Changes Over Time

**DOI:** 10.3389/fnins.2016.00211

**Published:** 2016-05-12

**Authors:** Sujoy Ghosh Hajra, Careesa C. Liu, Xiaowei Song, Shaun Fickling, Luke E. Liu, Gabriela Pawlowski, Janelle K. Jorgensen, Aynsley M. Smith, Michal Schnaider-Beeri, Rudi Van Den Broek, Rowena Rizzotti, Kirk Fisher, Ryan C. N. D'Arcy

**Affiliations:** ^1^Faculty of Applied Science, School of Engineering Science, Simon Fraser UniversityBurnaby, BC, Canada; ^2^NeuroTech Lab, Simon Fraser University and Fraser Health AuthoritySurrey, BC, Canada; ^3^Health Sciences and Innovation, Surrey Memorial Hospital, Fraser Health AuthoritySurrey, BC, Canada; ^4^Biomedical Physiology and Kinesiology, Faculty of Science, Simon Fraser UniversityBurnaby, BC, Canada; ^5^Sports Medicine Center, Mayo ClinicRochester, MN, USA; ^6^Department of Psychiatry, Icahn School of Medicine at Mount SinaiNew York, NY, USA; ^7^Joseph Sagol Neuroscience Centre, Sheeba Medical CentreRamat Gan, Israel; ^8^HealthTech Connex Inc.Surrey, BC, Canada

**Keywords:** clinical neuroscience, ERPs, evoked potentials, vital signs, neurology

## Abstract

Clinical assessment of brain function relies heavily on indirect behavior-based tests. Unfortunately, behavior-based assessments are subjective and therefore susceptible to several confounding factors. Event-related brain potentials (ERPs), derived from electroencephalography (EEG), are often used to provide objective, physiological measures of brain function. Historically, ERPs have been characterized extensively within research settings, with limited but growing clinical applications. Over the past 20 years, we have developed clinical ERP applications for the evaluation of functional status following serious injury and/or disease. This work has identified an important gap: the need for a clinically accessible framework to evaluate ERP measures. Crucially, this enables baseline measures before brain dysfunction occurs, and might enable the routine collection of brain function metrics in the future much like blood pressure measures today. Here, we propose such a framework for extracting specific ERPs as potential “brain vital signs.” This framework enabled the translation/transformation of complex ERP data into accessible metrics of brain function for wider clinical utilization. To formalize the framework, three essential ERPs were selected as initial indicators: (1) the auditory N100 (Auditory sensation); (2) the auditory oddball P300 (Basic attention); and (3) the auditory speech processing N400 (Cognitive processing). First step validation was conducted on healthy younger and older adults (age range: 22–82 years). Results confirmed specific ERPs at the individual level (86.81–98.96%), verified predictable age-related differences (P300 latency delays in older adults, *p* < 0.05), and demonstrated successful linear transformation into the proposed brain vital sign (BVS) framework (basic attention latency sub-component of BVS framework reflects delays in older adults, *p* < 0.05). The findings represent an initial critical step in developing, extracting, and characterizing ERPs as vital signs, critical for subsequent evaluation of dysfunction in conditions like concussion and/or dementia.

## Introduction

Vital signs such as heart rate, pulse oxygenation, and blood pressure are essential to monitoring and managing the health of various body systems. Yet there are no such vital signs identified for brain function—despite the clearly instrumental role such vital signs could play. Current clinical assessments for screening brain functional status relies largely on subjective, behavior-based measures, such as the Glasgow Coma Scale (GCS), to evaluate level of conscious awareness following brain injury (Teasdale and Jennett, 1974, pp. 81–84; Reith et al., 2016, pp. 89–94). However, subjective behavior-based tests of this nature have been reported to have misdiagnosis rates as high as 43% (Gawryluk et al., 2010, p. 11; Schnakers et al., 2009, p. 35). More detailed clinical evaluation of cognitive function and associated impairments are often reliant on neuropsychological assessment (Folstein et al., 1975, pp. 189–198; Lezak, 2004). These too are behavior-based measures, depending heavily on the patient's capacity to produce voluntary, on-demand motor and/or verbal responses to stimuli (Connolly and D'Arcy, 2000, pp. 31–47). Unfortunately, confounding factors, such as motoric and communicative limitations, often hamper greatly the clinical effectiveness for many of these measures.

Over the last 20 years, our group has demonstrated the critical need for a physiological, objective brain function assessment that utilizes event-related potentials or ERPs (Connolly et al., 1995, pp. 548–565; D'Arcy et al., 2003, pp. 662–672; Sculthorpe-Petley et al., 2015, pp. 64–72). ERPs are derived from long-standing electroencephalography (EEG; Pravdich-Neminsky, 1913, pp. 951–960). They can be recorded using minimal non-invasive scalp electrodes, combined with time-locked stimulation, to reflect target brain responses during information processing (Gawryluk and D'Arcy, 2010; Gawryluk et al., 2010, p. 11). EEG combines practical features of being accessible, available, low cost, and portable (Giacino et al., 2014, pp. 99–114), which makes the technology well suited for point-of-care applications (Gawryluk et al., 2010, p. 11). Work to date has demonstrated that ERPs can provide specific information across a spectrum of brain functioning, from low-level sensory to higher level cognitive processing (Luck, 2014). Moreover, ERPs have been shown to have robust diagnostic and prognostic capabilities (Morlet et al., 2000, pp. 198–206; D'Arcy et al., 2003, pp. 662–672; Kotchoubey et al., 2005, pp. 2441–2453; Wijnen et al., 2007, pp. 597–605; Vanhaudenhuyse et al., 2008, pp. 262–270; Daltrozzo et al., 2009, pp. 53–62).

In recent years, clinical ERP integration has focused on developing rapid, automated approaches in order to successfully utilize key ERPs that can be robustly recorded at the individual-level. The initial effort focused on developing a rapid evaluation framework for neurological status after severe acquired brain injuries, called the Halifax Consciousness Scanner (HCS; D'Arcy et al., 2011, pp. 750–754). The HCS was developed to examine the presence or absence of five key ERP responses linked to sensation (N100), perception (mismatch negativity, MMN), attention (P300), memory for one's own name (early negative enhancement, ENE), and semantic speech processing (N400). These ERPs were validated across a large sample of healthy individuals and clinically applied in neurological status assessment (Sculthorpe-Petley et al., 2015, pp. 64–72; Fleck-Prediger et al., 2014). However, it has become increasingly clear that mere assessments of presence or absence of a particular ERP does not fundamentally address the need to measure healthy individual brain function over time. Effective longitudinal monitoring of brain functional changes requires the establishment of individual functional “baselines” of brain vitality prior to conditions of dysfunction. Specifically, there is no framework for establishing and monitoring well established ERPs that can serve as indicators for an individual's healthy brain function, in spite of the evidence for relatively stable within-subject variance over time (Williams et al., 2005, pp. 1605–1630; Cassidy et al., 2012, pp. 659–664). This gap is essential to address in order to successfully assess the significance of any ERP-related change in which questions arise about possible dysfunction, which are increasingly arising as a potential application and common challenge in the evaluation of, for example, concussion and/or dementia.

Accordingly, the objective of the current study was to begin developing an initial framework to translate/transform ERPs into practical and accessible brain vital signs. The conceptual development of such a framework required a systematic process anchored to other existing vital sign frameworks. Therefore, the current paper is divided into two main sections: (1) a proposed conceptual framework for brain vital signs; and (2) a first step evaluation of practical implementation in a test sample of healthy adults across the lifespan.

I. Brain vital sign framework: As with other vital signs, a potential brain vital sign framework must satisfy some fundamental requirements: (1) the responses should be EEG hardware platform independent; (2) each response should be extensively characterized within the literature; (3) responses should be recorded reliably within healthy adult individuals^1^; (4) they should be accessible to normative data comparisons for essential response characteristics (e.g., amplitudes and latencies); and (5) importantly, the responses should be translated into a clinically accessible framework, which can be readily communicated.

**Figure 1:**
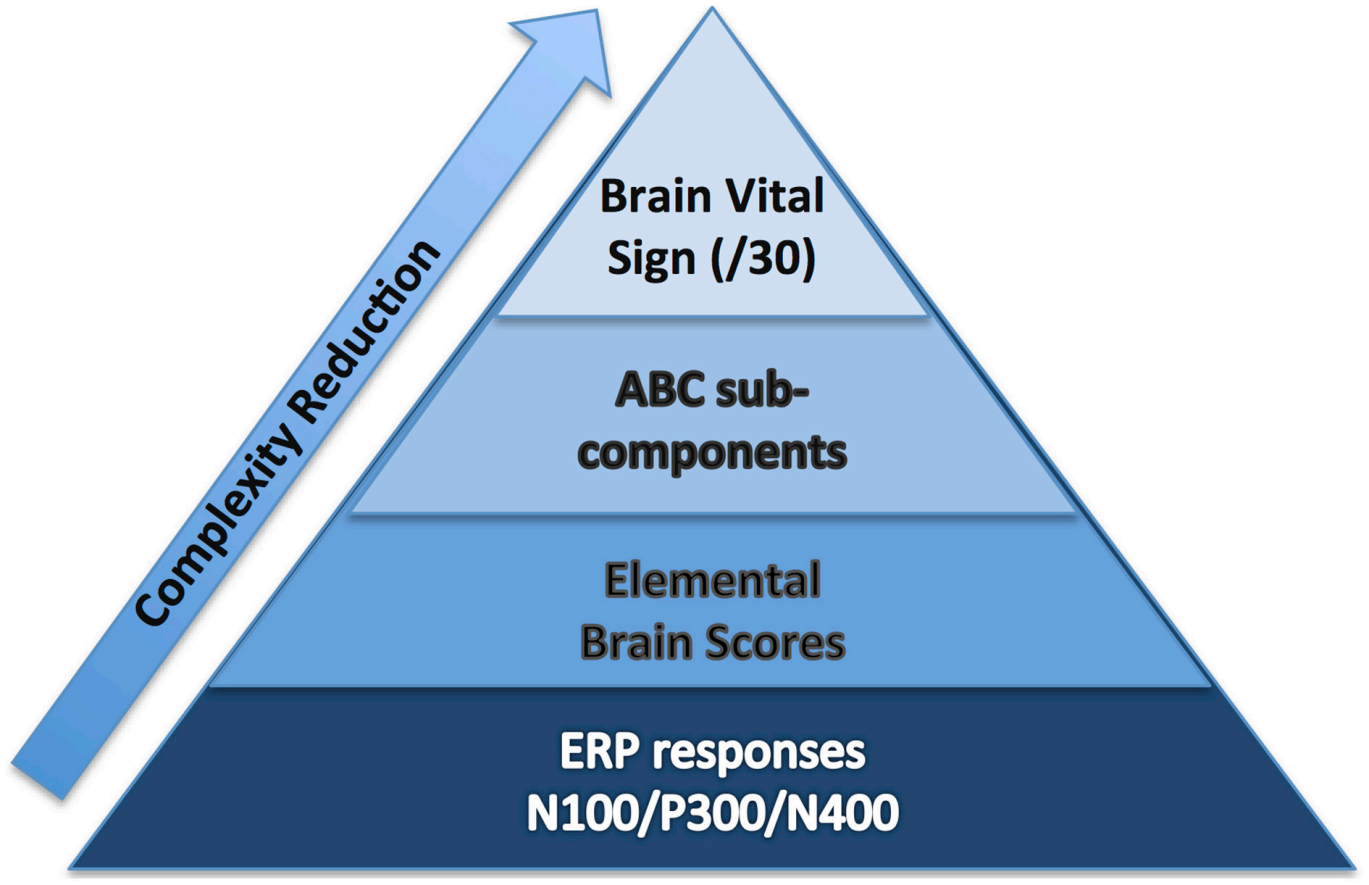
Brain vital sign framework: (1) overall brain vital sign score: highest 30; (2) ABC break down into Auditory sensation, Basic attention, and Cognitive processing; and (3) Elemental Brain Scores linearly transformed from N100, P300, and N400 response amplitudes and latencies (3 responses^*^2 measures = 6 scores).

**Figure 2:**
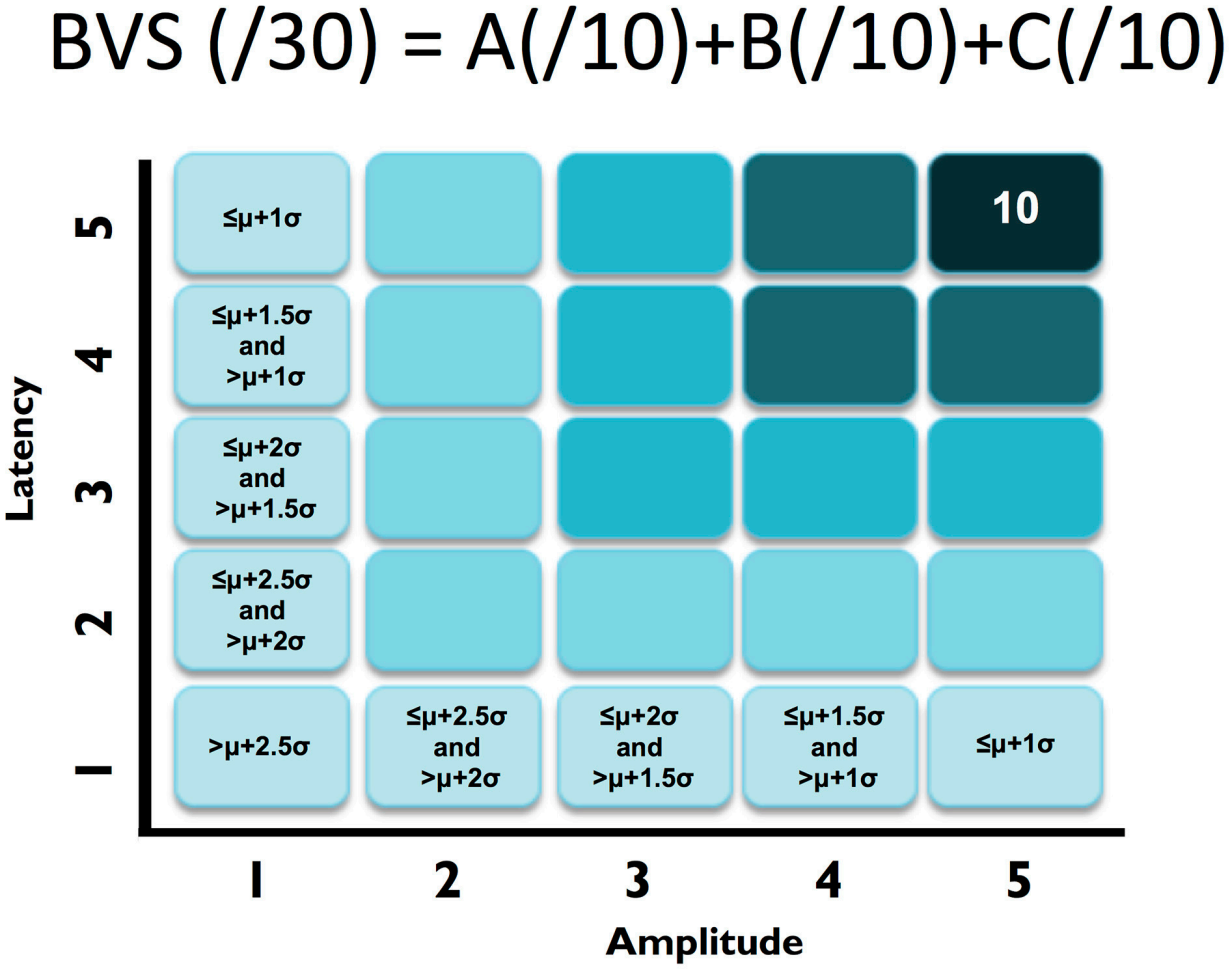
**ABC breakdown demonstrating graded measures**. Calculation shown for BVS sub-components “A”. Similar calculations undertaken for “B” and “C”.

To start, we selected well-established sensation-to-cognition ERPs^2^ : the N100, P300, and N400^3^. A complex-to-basic pyramidal approach provided an overview of the translation from technical ERP nomenclature to easy to communicate brain vital signs (Figure 1). Sub-scores reflective of specific brain functions were derived from the mean and standard deviations (Figure 2). Lastly, linearly transformed scores normalized to the best possible results for each amplitude and latency measure were created and referred to as elemental brain scores (EBS).

II. Practical implementation in healthy adults: To address implementation, the following steps were undertaken: (1) Hardware performance characterization was critical for platform independence. While some studies have compared hardware system performance for one time point analysis (Ries et al., 2014, pp. 10–20), analysis of performance over time was conducted to characterize instrument noise levels for longitudinal monitoring. (2) Stimulus sequence optimization was crucial to balance the trade-off between short testing times and highest possible response signal-to-noise ratio (SNR). (3) Response extraction and identification required an expert-independent, quality-checked approach. (4) Response results then required translation/transformation into the brain vital sign framework for interpretation and reporting. (5) A test sample of ERP data, with an age-related difference comparison embedded, provided initial validation across the adult life span (i.e., younger vs. older adults).

The current study utilized a healthy sample data set to test three hypotheses: (1) The three ERPs would be detectable at the individual level (Hypothesis 1); (2) Comparison between younger and older adults will show predictable age-related changes (Hypothesis 2); and (3) Because the brain vital sign framework is generated by applying a linear transformation to the raw ERP responses, we anticipate the pattern of age-related ERP changes would be preserved in the EBS results. With this initial step, it would then be possible to expand the brain vital sign framework into more extensive normative development along with applications in possible dysfunction related to conditions like concussion and dementia.

## Methods

### Characterizing and calibration of EEG hardware performance

Four candidate EEG systems (gNautilus and gMobiLab systems manufactured by g.Tec Medical Engineering, and two Enobio systems manufactured by Neuroelectrics) were evaluated in order to identify the most reliable hardware. Hardware evaluation used a 5-min known input calibration signal (“ground truth”), derived from a combination of sinusoidal waves with frequencies of 5, 10, 15, and 30 Hz (in MATLAB software). The test signal was delivered through the audio output port (at maximum volume setting) and recorded on 2 channels of the EEG systems as well as a Tektronix oscilloscope (model # 795-TBS1052B). Testing was conducted 2 times per day over 3 consecutive days (6 total).

Stability and reliability was assessed using inter-channel stability (correlation between channels at each time point of test), day-over-day stability (percentage change in peak voltage over the 3 days), peak-to-peak voltage recorded, and SNR (defined as ratio of sum of spectral power surrounding the 5, 10, 15, 30 Hz [“signal”] and 60 Hz [“noise”]). The g.Nautilus device, provided maximal SNR and had the best recording stability over the 3 days (average change 1.45% over 3 days, see Supplementary Material for full results), and was therefore utilized for subsequent human data collection.

Scalp-recorded ERPs were recorded from 3 midline electrodes (Fz, Cz, & Pz, embedded within a cap), insert earphones, g.Nautilus EEG acquisition hardware (bandpass: dc-250 Hz, 500 Hz sampling, 3 axis head motion accelerometers), and a portable computing platform. Four additional electrodes provide ground (forehead), reference (ear lobe) and eye monitoring (electro-oculogram, EOG).

Following signal amplification, conditioning, and digitization, the data were transmitted over Bluetooth link to the portable host computer. Time-stamping signals were sent from the host computer using custom-designed USB-to-TTL converter subsystem to mark stimulus presentation events. These TTL pulses were logged by the amplifier along with the EEG data and later used for signal averaging to derive ERPs.

### Stimulus sequence balancing SNR and short testing time

**Figure 3:**
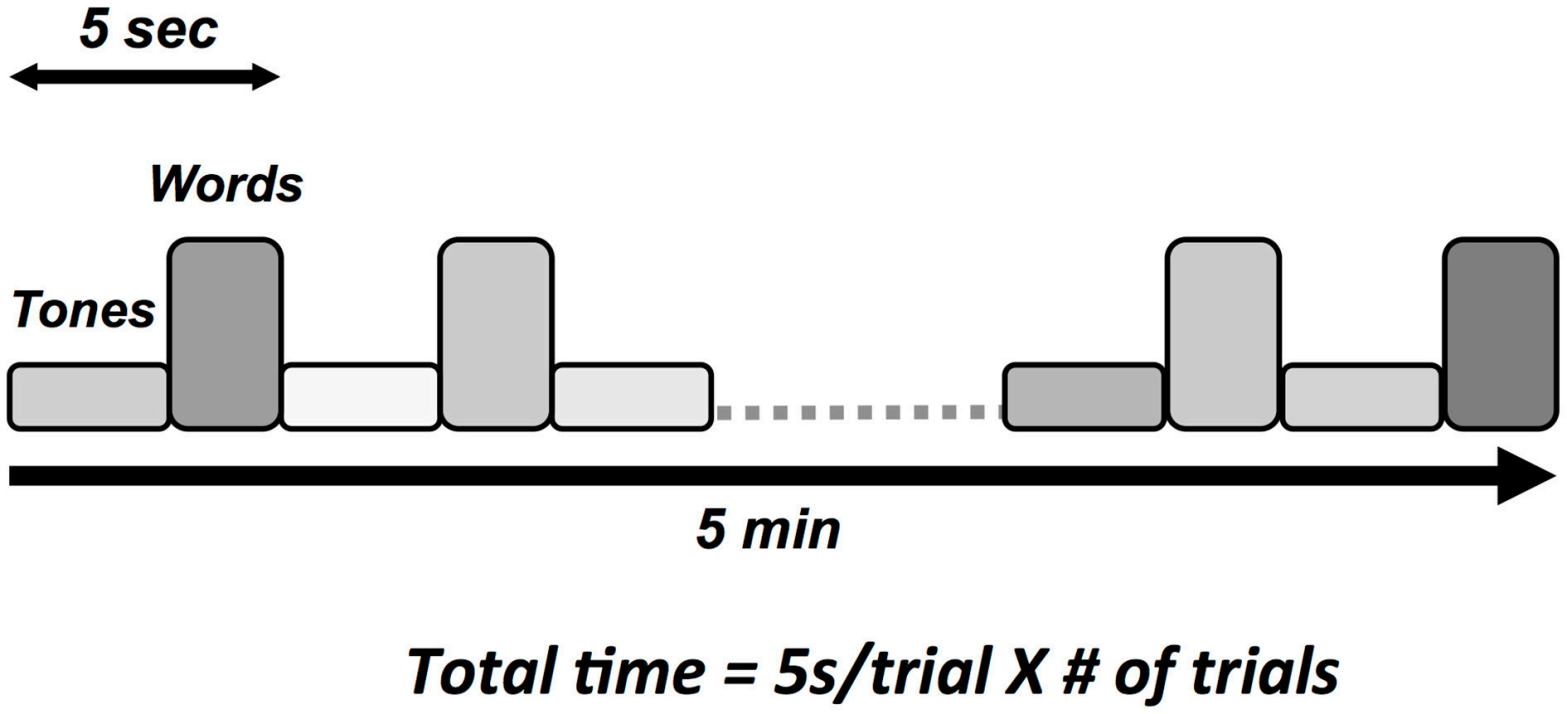
**Schematic illustration of auditory stimulus sequence consisting of words and tones**.

Auditory tone and spoken word pair stimuli were presented through the insert earphones. Tone stimuli elicited the N100 and P300 responses and spoken word pairs elicited the N400 (Figure 3). Tones (100 ms duration) were divided into standard (75 dB, 80%) and deviant (100 dB, 20%) conditions, with the N100 and the P300 derived from the deviant condition. Paired spoken words were divided into congruent prime pairs (e.g., bread-butter, 50%) and incongruent prime pairs (Romeo-table, 50%). The N400 was derived from the incongruent prime word pairs. The interlacing of tones and word pair stimuli enabled full optimization of near maximum trials per unit time (e.g., 5 s/stimulus cycle × 60 cycles = 5 min).

### ERP response elicitation, extraction and identification

EEG scans were conducted with minimal preparation compared to conventional EEG techniques (<5 min setup). Each participant was fitted with an elastic cap with embedded electrodes, and g.GAMMAsys electrode gel was injected at each location for conductivity. EOG channels were recorded using disposable Ag/AgCl electrodes on the supra-orbital ridge and outer canthus of the left eye. Skin-electrode impedances were maintained at <30kΩ impedance at each site. Acoustic stimuli were delivered binaurally through Etymotic ER4 insert earphones. Participants were instructed to pay attention to the auditory stimuli while maintaining visual fixation on a cross located 2.0 m away (black on white background). Three runs of the 5-min stimulus sequence were collected on each participant (approximately 15 min total run time).

Automated ERP pre-processing used established methods, including spectral filtering, segmentation, baseline correction, and conditional averaging (Luck, 2014). Signal-to-noise optimizations include ocular correction to remove eye artifact, jittered stimulus timing to minimize potential alpha contamination, and artifact de-noising using pattern recognition. ERP processing parameters were as follows: 1–20 Hz bandpass filter, 60 Hz notch filter, −100–900 ms epoch length for segmentation relative to stimulus onset. ERP response identification was undertaken through a template matching process in which N100, P300, and N400 peaks were identified by specifying expected polarity within expected temporal ranges (Marchand et al., 2002, pp. 1715–1722). Each ERP response value was measured as peak-to-peak measure relative to the preceding peak of opposite polarity.

Machine learning methods such as support vector machine (SVM), allow training of two-category classifiers to distinguish contrasting experimental conditions (see Parvar et al., 2014, pp. 1–12; Sculthorpe-Petley et al., 2015, pp. 64–72). The best results were obtained using single run, trial-averaged data from all three-electrode sites as inputs to the SVM with a radial kernel. Ninety Percent of the available data were randomly selected to train a two-category classifier to distinguish between the two stimulus conditions for each experiment (i.e., standard vs. deviant tones for N100 & P300, congruent vs. incongruent words for N400). The trained classifier was then applied to the remaining 10% of datasets to test the accuracy of classification. Under 10-fold cross-validation, this process is repeated 10 times such that the classifier is trained and tested on all available data. The total instances of correct group classification (ex. number of correct standard and deviant classifications) relative to the total number of classifications provide an accuracy number. Standard statistical measures including true positive (TP), false positive (FP), sensitivity, and specificity are derived from the confusion matrix. The SVM analyses were further verified using non-parametric permutation statistics to assess if the observed performance could be obtained by chance ((Golland and Fischl, 2003), pp. 330–341). This involved randomly redistributing the class labels in the training sets and observing the performance of the new SVM solution. After 1000 permutations, the observed classification accuracies were used as a null distribution against which the significance of the true SVM solution was determined.

### Translation/transformation of ERP responses to brain vital sign framework

First, similar to neuropsychology assessment, a total brain vital sign score of 30 was defined to represent the most basic result: all responses fall with the normative range, bounded by standard deviation. The highest level of brain vital sign framework combines all three ERP peak amplitude (μV) and latency (ms) measures, ranked in terms of standard deviations from the mean (M/SDs), into one composite score of 30. The total brain vital sign score of 30 reflects overall healthy brain processing^4^. The total scores for each participant were generated by comparing the amplitude (X) and latency (L) measures of each of the 3 components to the normative database, with scoring criteria determined using the mean (μ) and standard deviation (σ) of the corresponding measures in the normative database. Details are shown in Table 1.

**Table 1:** **BVS scoring criteria for the three ERP components**.

**P300**		**N400 & N100**	
**Amplitude (X)/Latency (L)**	**BVS Score**	**Amplitude (X)/Latency (L)**	**BVS Score**
X > μ −σ	5	X < μ + σ	5
L < μ + σ		L < μ + σ	
μ − 1.5σ < X < μ −σ	4	μ + 1.5σ > X > μ + σ	4
μ + 1.5σ > L > μ + σ		μ + 1.5σ > L > μ + σ	
μ − 2σ < X < μ − 1.5σ	3	μ + 2σ > X > μ + 1.5σ	3
μ + 2σ > L > μ + 1.5σ		μ + 2σ > L > μ + 1.5σ	
μ − 2.5σ < X < μ − 2σ	2	μ + 2.5σ > X > μ + 2σ	2
μ + 2.5σ > L > μ + 2σ			
		μ + 2.5σ > L > μ + 2σ	
X < μ − 2.5σ	1	X > μ + 2.5σ	1
L > μ + 2.5σ			
		L > μ + 2.5σ	

A standard clinical scheme of “ABC” was implemented for the breakdown of individual responses, with the N100 as an indicator for Auditory sensation (A) (Davis, 1939, pp. 494–499); the P300 as an indicator for Basic attention (B) (Sutton et al., 1967, pp. 1436–1439); and the N400 as an indicator for Cognitive processing during speech perception (C) (Kutas and Hillyard, 1980, pp. 203–205). Within the ABC scheme 5 points for amplitude and latency each were awarded. In addition to establishing a healthy brain vital sign range (*A* = 10, *B* = 10, *C* = 10; Total 30), it was also possible to derive metrics for monitoring ABC amplitude and latency changes over time. Amplitude and latency metrics for ABC were used to calculate 6 elemental brain scores (EBS). Each EBS was normalized to the best possible response measurement. Therefore, for each EBS, it is possible to rank ABC amplitude and latency results relative to the largest normative ERP response amplitude and shortest normative ERP response latency, with scores ranging from 0 to 1. A score of 1 matched the outer bounds for best possible measurement.

Mathematically, EBS measures can be expressed as shown in equations 1 and 2 below:

(1) $$\text{Score}=\text{1}-\text{abs }(\frac{\text{M}-\text{best}}{\text{max}-\text{min}})$$

(2) $$\text{Score}=\text{1}-\text{abs }(\frac{\text{best}-\text{M}}{\text{max}-\text{min}})$$

Where, *M* is the mean value of the amplitude/latency, *max* is the maximum value and *min* is the minimum value and *best* is the “ideal” value that should be achieved. Best value can either be the max or the min value depending on whether the lowest or the highest value represents the ideal situation—generally for latency the lowest (smallest) value represents faster (better) processing, whereas for amplitude the highest positive value or lowest negative value is thought to represent ideal processing.

Equation (1) was utilized for N100 and N400 amplitude and latency as well as P300 latency, whereas Equation (2) was used for P300 amplitude. All EBS calculations were undertaken using an existing database of 100 healthy controls (Sculthorpe-Petley et al., 2015, pp. 64–72) containing information about N100, P300 and N400 components. To account for outliers in the normative database, all data values were ranked, and the interquartile range between 75th and 25th percentiles calculated. Extremity thresholds were determined by calculating the points corresponding to 1.5-times the interquartile range above the 75th percentile, and 1.5-times the interquartile range below the 25th percentile. Data points beyond the boundary formed by these thresholds were excluded as outliers from the normative database prior to BVS and EBS extraction. This process resulted in the removal of 6 participants (out of 100) from the N100 amplitude database, 2 participants from the N100 latency database, 1 participant from the N400 amplitude, 6 participants from the P300 amplitude database and 1 participant from the P300 latency database. No participants were excluded from the N400 latency database.

### Initial validation across the healthy adult lifespan

Sixteen (16) participants ranging in age from 22 to 82 years were recruited (46.81 ± 22.14, 8 females). A bimodally distributed sample was selected across lifespan, with 8 in the 20–35 year-old range (26.13 ± 4.00, 4 females) and 8 in the 50–85 year-old range (67.5 ± 11.25, 4 females). Participants had no history of neurological problems or psychoactive medications. All individuals were fluent in English and had normal or corrected-to-normal hearing. EEG noise compromised data from 2 participants. Furthermore, to ensure analyses used a minimum of a 20-year age separation with controlled age-appropriate cognitively matched samples, data from 2 participants were not included (1 from each sub-sample). The final matched analysis therefore included 6 in the 20–30 year range and 6 in the 50–85 year range. Research Ethics Boards at Simon Fraser University and Fraser Health Authority approved the study.

Each participant underwent neuropsychological screening along with EEG/ERP testing using Mini Mental State Exam (MMSE) (Folstein et al., 1975, pp. 189–198) and Montreal Cognitive Assessment (MoCA) (Nasreddine et al., 2005, pp. 695–699). MMSE examines 5 areas of cognition (orientation, registration, attention and calculation, recall, and language), with scores below 23 suggestive of cognitive impairment (maximum score 30; Folstein et al., 1975, pp. 189–198). MoCA examines a multitude of high-level cognitive functions (e.g., short-term memory recall, delayed recall, visuospatial abilities, working memory, and language etc.).

ERP results were divided into the two groups (20–30 and 50–85 age ranges). Quantitative group-level ERP response characteristics were compared using two-tailed independent samples *t*-test. Results are presented as mean ± SD. Moreover, to assess the performance of the expert-independent method (SVM) across the age ranges, a sub-analysis was undertaken for each group to compare and contrast any performance differences.

ERP responses were also transformed into the brain vital sign framework, generating an overall brain vital sign score and the 6 EBS scores each participant. EBS scores were compared at the group-level. Normality was assessed using the Shapiro-Wilk W test. Only the EBS measures for amplitude in the “C” component of the framework did not pass the normality test, and they were therefore compared using the Wilcoxon test. All others were compared using two-tailed independent-samples *t*-test. Results are presented as mean ± SD.

## Results

### Participant cognitive status evaluation

Participant characteristics and MMSE/MoCA scores are presented in Table 2. Both the younger (age 20–30) and older (age 50–85) groups scored in the healthy range. All individuals in the younger group obtained full scores (30/30) for both MMSE and MoCA. Participants in the older group scored 30 for MMSE and 29.3 ± 0.5 for MoCA.

**Table 2:** **Sample characteristics and cognitive test scores**.

	**Age 20–30**	**Age 50–85**
Sample Size (n)	6	6
Education (years)	18.3 ± 1.9	17.8 ± 5.6
MMSE (/30)	30	30
MoCA (/30)	30	29.3 ± 0.5
Sex (M:F)	1:1	1:2

### ERP response extraction and expert-independent identification

**Figure 4:**
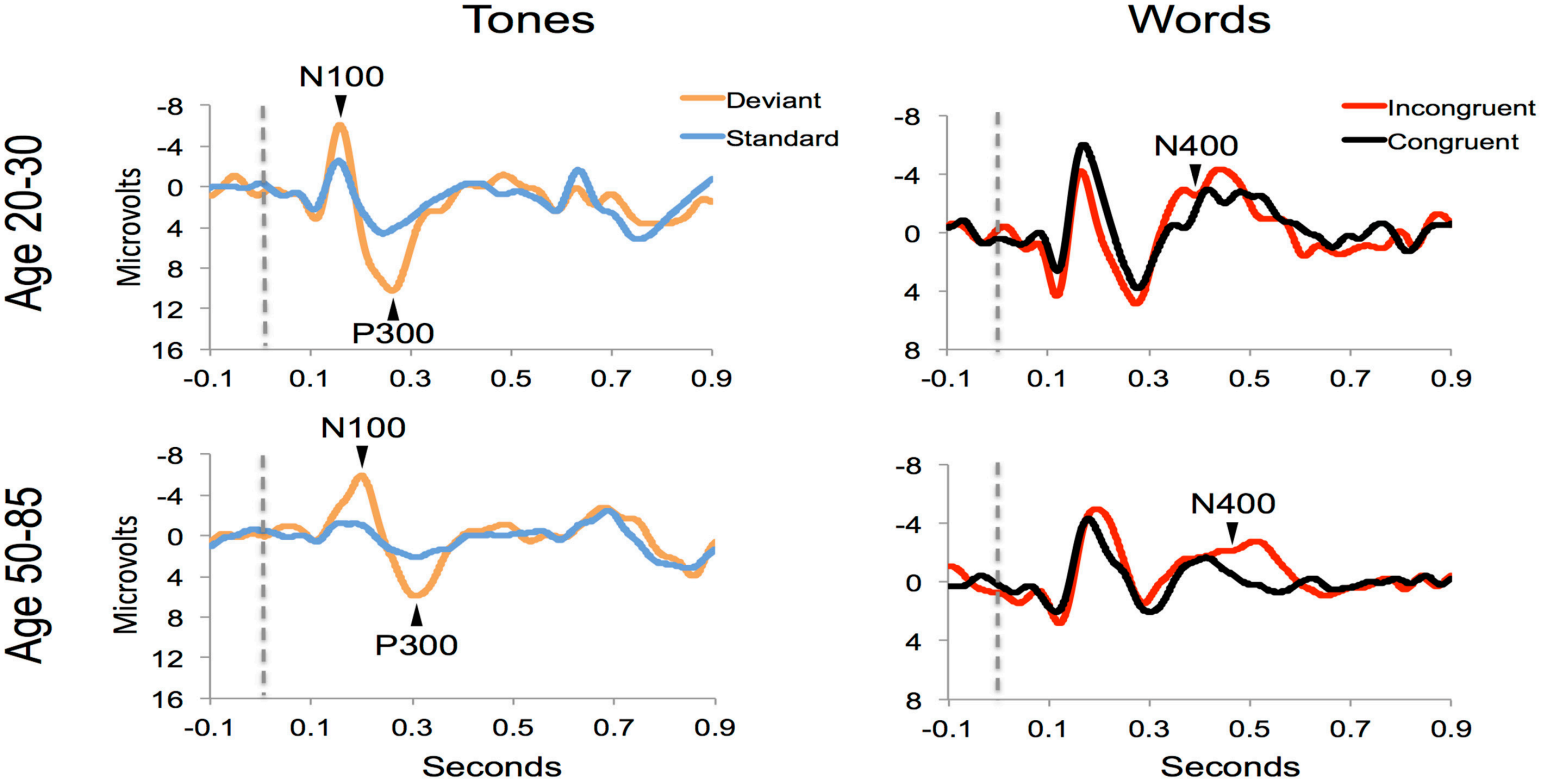
**ERP waveforms for a representative participant in the younger (age 20–30, participant age = 30) and middle-aged/older (age 50–85, participant age = 60) age ranges**. Data were averaged across 3 runs.

**Figure 5:**
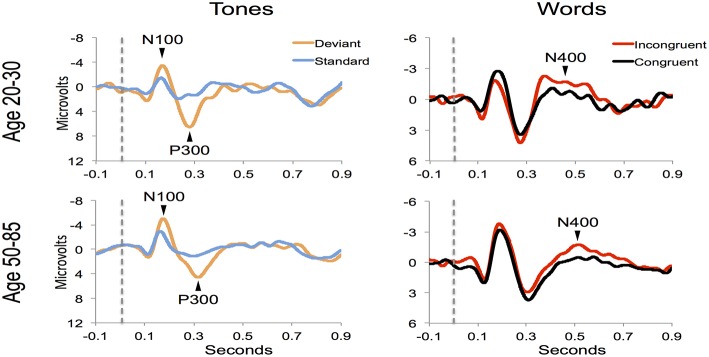
**ERP waveforms for group averages in the younger (age 20–30) and middle-aged/older (age 50–85) age ranges**.

Figure 4 shows the ERP components evoked using the stimulus sequence in representative individuals for the 20–30 and 50–85 age ranges. The N100 component was elicited during Auditory sensation (−6.74 ± 2.13 μV). The P300 was elicited during Basic attention to deviant tones (10.72 ± 2.66 μV). The N400 was elicited during Cognitive processing of semantically incongruent word pairs (−5.09 ± 2.67 μV). All components were present in all participants. Figure 5 presents group-averaged ERP results.

ERP response results indicate that the trained SVM classifier successfully identified predicted response differences. For the P300, the SVM classification included deviant vs. standard tones. The individual-level accuracy of P300 classification is 98.96% across all ages, with 0.98 sensitivity and 1.00 specificity (Table 3). For the N400, the SVM classification included incongruent vs. congruent word pairs. The individual-level classification accuracy for N400 is 86.81% across all ages, with 0.84 sensitivity and 0.90 specificity (Table 3). Permutation analysis verified the accuracy of the SVM classification for P300 (*p* < 0.001) and N400 (*p* = 0.05).

**Table 3:** **SVM classification for P300 and N400**.

	**P300**	**N400**
Accuracy	98.96%	86.81%
True positive	0.98	0.84
False positive	0.00	0.10
Sensitivity	0.98	0.84
Specificity	1.00	0.90

### Translation to the brain vital sign framework

The participant responses were successfully translated into the brain vital sign framework. Relative to norms, the representative participants in Figure 5 both scored full 30, allocated the maximum 10 for each of the A, B, and C components. All individuals achieved scores of 30. EBS scores were also calculated: (A) amplitude (0.56 ± 0.17) and latency (0.41 ± 0.25) for the N100 using *N* = 99 norms; (B) amplitude (0.59 ± 0.11) and latency (0.59 ± 0.14) for the P300 using *N* = 100 norms; and (C) amplitude (0.50 ± 0.24) and latency (0.36 ± 0.16) for the N400 using *N* = 100 norms. As anticipated, each of the EBS measures straddled the 50th percentile mark (= 0.5, representing average performance) within the mean ± 1 standard deviation segment.

### Initial validation across the healthy adult lifespan

**Figure 6:**
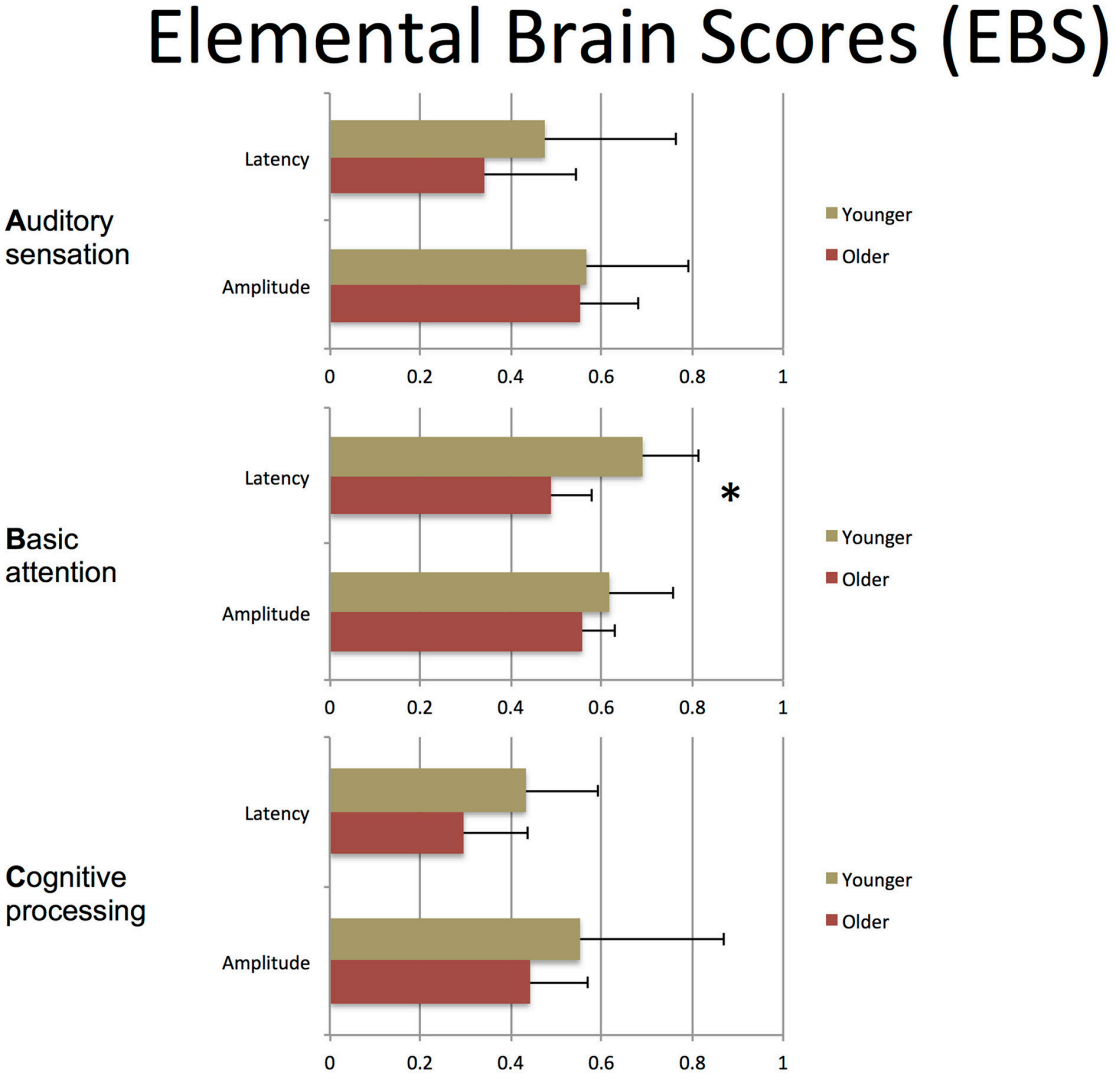
**EBS for group-level comparison**. Mean ± SD. ^*^p < 0.05 across groups.

Table 3 presents quantitative group-level component ERP response characteristics. Table 4 shows that P300 latencies increased significantly between younger and older groups (*p* < 0.05), with a similar trend for the N400 latencies (*p* = 0.07). Table 5 shows that SVM classification undertaken separately for the younger and older age groups, showed comparable results between the two groups. Figure 6 and Table 6 show group-level EBS scores between younger and older groups. Similar to P300 latency measures, the corresponding EBS score (“B” latency) demonstrated a significant group difference (*p* < 0.05) between old and young. Additionally, the “C” latency EBS also showed a trend (*p* = 0.07) for group differences, again in agreement with the corresponding N400 latency trends.

**Table 4:** **Quantitative measures for group-level ERP characteristics**.

		**Age 20–30**	**Age 50–85**
P300	Amplitude (μV)	11.09 ± 3.39	10.36 ± 1.91
	Latency (ms)	276.00 ± 20.59	310.00 ± 15.02^*^
N400	Amplitude (μV)	5.93 ± 3.60	4.51 ± 1.00
	Latency (ms)	460.67 ± 65.11	516.67 ± 57.53

Mean ± SD.

* p < 0.05 between groups.

**Table 5:** **SVM classification comparisons between the two age groups**.

	**P300, Younger**	**P300, Older**	**N400, Younger**	**N400, Older**
Accuracy	99.31%	98.61%	86.11%	86.11%
TP	0.99	0.97	0.88	0.85
FP	0.00	0.00	0.15	0.13
Sensitivity	0.99	0.97	0.88	0.85
Specificity	1.00	1.00	0.85	0.88

TP, True Positive; FP, False Positive.

**Table 6:** **EBS values for group-level characteristics**.

		**Age 20-30**	**Age 50-85**
N100	Amplitude	0.57±0.22	0.55±0.13
	Latency	0.47±0.29	0.34±0.20
P300	Amplitude	0.62 ± 0.14	0.56 ± 0.07
	Latency	0.69 ± 0.12	0.49 ± 0.09^*^
N400	Amplitude	0.55 ± 0.32	0.44 ± 0.13
	Latency	0.43 ± 0.16	0.30 ± 0.14

Mean ± SD.

* p < 0.05 between groups.

## Discussion

The current study had two objectives: (1) to describe a conceptual framework for brain vital signs, which can provide an objective physiological evaluation of healthy baseline brain function; and (2) to conduct an initial practical evaluation in a test sample of healthy adults across the lifespan. The results demonstrated the successful detection of the three key ERPs at the individual level (Hypothesis 1), confirmed the expected pattern of age-related ERP changes (Hypothesis 2), and enabled the translation of ERPs into the brain vital sign framework (Hypothesis 3). Importantly, this provided the initial step toward a brain vital sign approach that preserves and simplifies the essential valuable ERP results, but enables practical, accessible “vital sign” attributes.

Robust individual level detection of ERPs like the N100, P300, and N400 has become possible through machine learning advances (Parvar et al., 2014; Sculthorpe-Petley et al., 2015). Indeed, even within the current small initial validation sample, the ERPs were successfully detected for individuals across the life span (Figures 4, 5). SVM-based analysis allowed expert-independent validation with high accuracy, sensitivity, and specificity. SVM-based methods are generally considered extremely well suited for use in biomedical data due to their ability to deal with sparse learning scenarios (Yeo et al., 2009, pp. 115–124). Traditionally, SVM-based techniques for ERP are restricted to within-subject training and classification for brain machine interface applications (Parvar et al., 2014, pp. 1–12). By contrast, in the current application the SVM-based methods involved between-subject training and classification, further demonstrating potential for robust clinical applications. Moreover, permutation analysis based verification of performance provides further confidence regarding the robustness of these approaches.

As an initial validity check, predictable age-related changes in ERPs were examined and verified within the brain vital sign framework. To demonstrate the relative sensitivity differences between subjective behavioral tests and objective physiological measures, standard mental status assessments were compared to that of the ERP results. Results from MMSE and MoCA were both in the healthy range for the younger (age 20–30) and older (age 50–85) groups. While the ERP results generally matched this pattern, it was possible to show subtle age-related P300 latency delays (*p* = 0.008) in older adults, consistent with previous studies (Braverman and Blum, 2003, pp. 124–139). A similar trend was observed for N400 latency delays (*p* = 0.07). Thus, while both behavior and brain—based testing showed intact cognitive status, only the ERP evidence showed enhanced sensitivity to age-related changes in healthy brain function. Future work will further characterize standard factors in larger normative samples. These include characterization of aging related confounds, effects of education levels, impact of concurrent changes in other vital signs such as heart rate and blood pressure, and correlations between specific EBS components and traditional behavioral measures. Similarly, planned future work will also explore the opportunity to include both resting state as well as other stimulus-related brain response measures (such as event related spectral perturbations) into the BVS framework.

To translate ERP results into the brain vital sign framework, we applied a linear transformation to reduce complexity and create a standard clinical schematic of ABC: (A) N100 = Auditory sensation; (B) P300 = Basic attention; and (C) N400 = Cognitive processing (Figure 1). Brain vital sign scores were then derived through comparison to the mean and standard deviation of the normative data. All participants showed an overall brain vital sign score of 30, derived from perfect 10-point ABC sub-scores (Figure 2). This component provided a normative evaluation for healthy brain function. As an initial development and to retain applicability over a wide range of potential dysfunction, all components were weighted equally in this framework. Future work may create variations/improvements that weigh the components differently for applications in specific disorders.

To transform ERP results into measurements of individual changes over time, the amplitude and latency measurements for all three responses were converted into 6 elemental brain scores (EBS: 3 responses × 2 measurements). Importantly, the EBS transformation involved a normative comparison against the best possible measurement, resulting in scores ranging from 0 to 1. During initial validation, EBS transformation preserved the pattern of age-related changes, with significant change in the “B” component latency (*p* = 0.004) and a similar trend in “C” component latency (*p* = 0.07).

The justification for a brain vital sign framework is strongly within the need for a practical and objective physiological measure of healthy brain function, combined with the capability for portable EEG/ERPs to meet the practical requirements and utilize well-established neural responses (i.e., studied extensively for 35–70 years). The challenge has related to translating/transforming ERPs to begin addressing the clinical requirements for vital signs.

Accordingly, the current study represents only an initial development effort, with a number of steps and caveats remaining: (1) the initial validation used a relatively small sample size, with further validation work currently being conducted; (2) the critical need for hardware platform independence remains to be systematically examined in order to understand differences between EEG acquisition systems; (3) the development of standardized normative databases represents an on-going improvement and refinement; (4) the continuing development of analyses to characterize sensitivity, specificity, reliability, and other standard metrics are needed; and (5) more comprehensive evaluations anchored to standard vital sign developmental approaches must also be conducted. Nonetheless, the ability to move beyond the traditional and heavily expert-dependent ERP research setting to a more clinically-oriented brain vital sign framework allows for a systematic method of assessing healthy brain function. The current study provides an initial demonstration of the framework, but the small sample size necessitates that the results should be further validated in a larger sample. Furthermore, it should be noted that there are several approaches available for eliciting the ERP components. We have demonstrated one approach that we believe makes the oddball discrimination task easier in order to maximize applicability across age groups and brain functional status. Establishing a baseline measurement approach for healthy brain function is critical, particularly when questions of dysfunction arise due to conditions such as concussion and dementia. This study represents the initial steps toward such an approach.

## Conclusion

Clinical evaluations of healthy brain functioning is moving from indirect subjective behavior-based tests, to objective, physiological measures of brain function, such as those derived from ERPs. We have previously demonstrated the essential role for clinical ERPs to evaluate functional status following serious injury and/or disease. The current study addressed an important gap: the need for a clinical-accessible brain vital sign framework that utilizes well-established ERPs. As an initial step, the framework was used to evaluate healthy brain function across the life span. The findings confirmed the ERPs at the individual level, verified predictable age-related differences, and demonstrated successful linear transformation to create the brain vital sign framework.

## Author contributions

Conceptualization and study design: SG, CL, XS, and RD. Literature search: SG, CL, and RD. Data collection: SG, CL, SF, LL, and GP. Analysis planning: SG, CL, XS, RV, RR, KF, and RD. Data analysis: SG, CL, and LL. Result presentation: SG, CL, XS, and RD. Analysis outcome verification: SG, CL, XS, and RD. Result interpretation: All authors. Manuscript preparation: SG, CL, and RD. Critical editing and approval of submission: All authors.

## Funding

This work was supported in part by Mathematics of Information Technology and Complex Systems (MITACS, Grant #IT03240), National Sciences and Engineering Council Canada (NSERC, Grant #298457-2009), and Canadian Institutes for Health Research (CIHR, Grant #CCI-109608).

### Conflict of interest statement

Several of the authors are associated with HealthTech Connex Inc. which may qualify them to financially benefit from the commercialization of a NeuroCatch™ platform capable of measuring brain vital signs.

## Footnotes

^1^Most ERP responses are also well characterized across brain development, but require increasing reliance on age specific norms and caveats that are not discussed here.

^2^While the main focus is on auditory ERP responses, all three are present across both auditory and visual modalities.

^3^Note that the mismatch negativity (MMN; Duncan et al., 2009) may also be considered a potential ERP response for a brain vital sign framework. As the MMN can also be derived from the same auditory stimuli that elicit the N100/P300, it remains possible candidate for future expansion.

^4^Note that the initial SD cut-off can be customized and optimized in accordance with the enhanced development of normative databases.
