# Supplementary material for: Developing Brain Vital Signs: Initial Framework for Monitoring Brain Function Changes Over Time
Source: Front Neurosci. 2016 May 12;10:211. doi: 10.3389/fnins.2016.00211 (PMC4867677; doi:10.3389/fnins.2016.00211)
Supplement: Supplementary file 1 [file DataSheet1.DOCX]

Supplementary Material

Developing brain vital signs: Initial framework for monitoring brain function changes over time

S. Ghosh Hajra, C. C. Liu, X. Song, S. Fickling, L. E. Liu, G. Pawlowski, J. K. Jorgensen, A. M. Smith, M. Schnaider-Beeri, R. van den Broek, R. Rizzotti, K. Fisher, R. C. N. D’Arcy*

*** Correspondence:** Ryan C.N. D’Arcy Ph.D., NeuroTech Lab, Barham Pavillion, Surrey Memorial Hospital, 13750 96 Avenue, Surrey, British Columbia, V3V 1 Z2, Canada.

rdarcy@sfu.ca

# Supplementary Figures


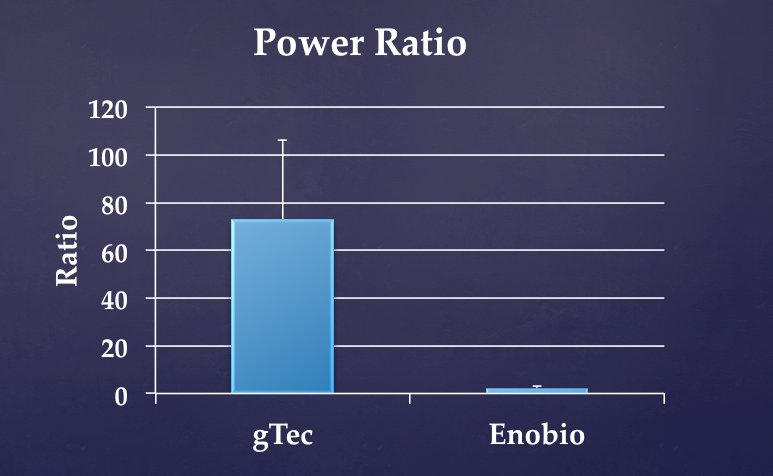


**Supplementary Figure 1.** g.Nautilus systems (manufactured by g.tec) have improved ratio of signal to noise compared to the Enobio system (manufactured by Neuroelectrics). The power ratio was calculated as sum of power in the 5, 10, 15 and 30Hz regions (‘signal’) and the power surrounding 60Hz (‘noise’) with equalized numbers of frequency bands in numerator and denominator. Only systems with 500Hz sampling frequency among the tested systems are shown here.

**Supplementary Figure 2.** The g.Nautilus system has better consistency over days of testing. Again, only systems with the same sampling frequency (500Hz) are shown here. Average change over 3 days for the g.Nautilus systems was 1.45%.
